# Cognitive function in normotensive elderly adults: a population-based cross-sectional study in rural China

**DOI:** 10.3389/fmed.2026.1756221

**Published:** 2026-03-09

**Authors:** Changqing Zhan, Qiao Wang, Yingnian Chen, Na Pan, Wenyu Wang, Xueping Lu, Xinling Xie

**Affiliations:** 1 Department of Neurology, The Second People's Hospital, Wuhu, China; 2 Department of Pathology, The Second People's Hospital, Wuhu, China; 3 Department of neurological rehabilitation, Anhui Wannan rehabilitation hospital, Wuhu, Anhui, China; 4 Graduate School of Bengbu medical university, Bengbu, Anhui, China; 5 Graduate School of Wannan Medical College, Wuhu, China

**Keywords:** BMI, cognitive, FBG, MMSE, risk factors

## Abstract

**Introduction:**

Worldwide, the number of people with cognitive disorders is rapidly increasing. The risk factors for cognitive dysfunction in normotensive elderly individuals remain unclear. This study explored cognitive function and related risk factors in a rural elderly population in Tianjin, China.

**Methods:**

Participants were recruited from the Tianjin Brain Research Institute. Cognitive function was assessed using the Mini-Mental State Examination (MMSE). Multivariate logistic and linear regression analyses were performed using SPSS (version 27.0), with statistical significance set at *P* < 0.05.

**Results:**

A total of 386 participants (191 males, 195 females; mean age 65.88 years) were included. Multivariate analysis revealed that gender, age, and body mass index (BMI) were significantly associated with MMSE scores. Women scored 2.40 points lower than men (95%CI: −3.78, −1.02, *P* < 0.001). Individuals aged ≥75 scored 3.18 points lower than those aged 60–64 (95%CI: −4.88, −1.49, *P* < 0.001). BMI was positively correlated with MMSE scores, increasing by 0.18 points per unit increase in BMI (95%CI: 0.03, 0.33, *P* = 0.019). Subgroup analysis exploratory results suggested that age may be associated with cognitive impairment in women, while BMI may show a positive correlation with MMSE scores in men. Among participants aged ≥75 years, alcohol status may benefit cognition; triglycerides generally exhibited a risk trend but may show an inverse association in the 70–74 age group. Blood glucose levels showed no significant effect on cognition (*P* > 0.05).

**Conclusion:**

Older age, female gender, and lower BMI are associated with lower MMSE scores in normotensive elderly individuals. Strengthening community screening and education for older women and men with low BMI is essential.

## 1 Introduction

Worldwide, the number of people with cognitive disorders is rapidly increasing. In part, this is inextricably linked to the aging of the population, as dementia is a disorder of aging (1). In 2016, the prevalence of dementia was 10% in people aged 65 and over and 35% in people aged 90 and over in the United States (2). Alzheimer's Disease (AD) is the most common cause of dementia, worldwide (3). In 2018, the Alzheimer's Association International estimated that around 50 million people worldwide suffer from dementia, which is expected to triple by 2050, with two-thirds of them living in low-income and middle-income countries (4). And the median survival time after diagnosis of Alzheimer's disease dementia is 6.2 years (5). Dementia often has severe cognitive, behavioural and functional consequences, which not only endangers the life and health of patients, but also has a great impact on caregivers. Therefore, early prevention of dementia is of great importance to reduce the social and economic burden.

Previous studies indicated that the risk factors of cognitive dementia included old age, 1 less education, obesity, diabetes, hypertension, alcohol misuse, smoking (6). Hypertension has been widely confirmed as a risk factor for cognitive impairment, and the prevalence of cognitive impairment was markedly higher among individuals with hypertension than those normotensive (7–9). Whether diabetes or elevated fasting blood glucose (FBG) levels are associated with cognitive decline remains controversial. Some studies suggested that diabetes was associated with an increased risk of cognitive decline, and preventing chronic hyperglycemia may help maintain cognitive function (10–13). But other studies have shown that hyperglycemia was not significantly associated with cognitive impairment (14–16). Multiple studies have shown that being underweight or having a decreased BMI were a risk factor for cognitive impairment (17–20) and the association between BMI and cognition was different in different age groups (21). However, it is uncertain whether the elderly population with normal blood pressure has the same risk factors for cognitive impairment as the elderly population with hypertension. Few studies have investigated the risk factors for cognitive impairment in normotensive individuals, especially low-income elderly individuals. As a result, there is a lack of targeted prevention strategies for those population. Therefore, this study aimed to evaluate the risk factors for cognitive impairment in normotensive elderly adults in rural China, and to contribute to the development of more targeted prevention strategies for this population.

## 2 Materials and methods

### 2.1 Study population

**Figure 1:**
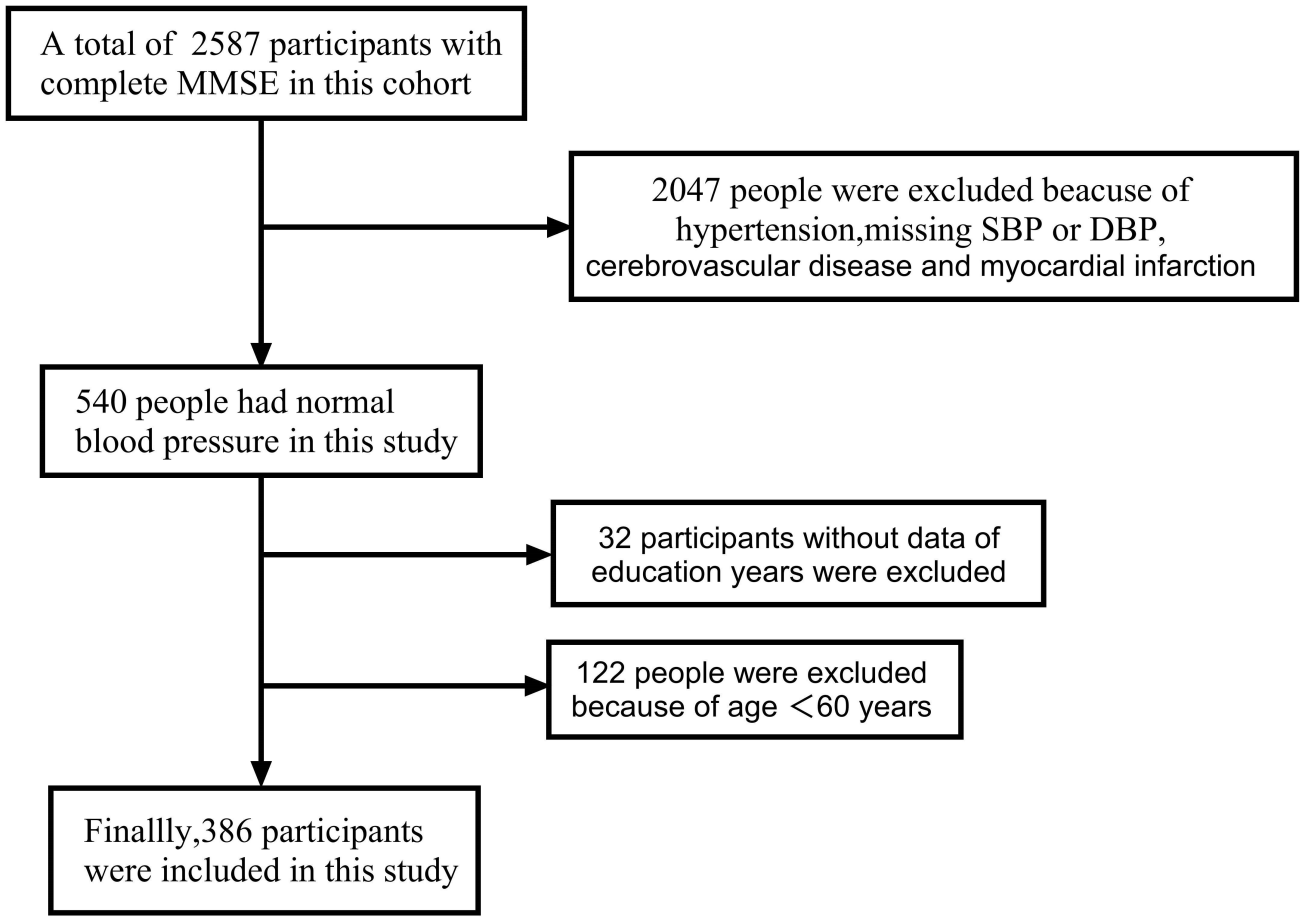
Flow chart of participant enrollment. During the 2012, 2019,and 2020 survey waves, 2,587 subjects who completed the MMSE were screened; 386 normotensive individuals aged ≥ 60 years were finally included.

The study is a cross-sectional study with subjects from the Tianjin Brain Research Institute, and data from 2012 to 2019, and 2020 were studied. A total of 2,587 subjects were included in this cohort, inclusion criteria included people aged 60 years or older with normal blood pressure with complete MMSE score data. People with a previous history of stroke, myocardial infarction, and hypertension were excluded. A total of 386 participants were included (Figure 1).

All participants provided informed consent at the start of the study. The study was approved by the Tianjin Medical University General Hospital and was conducted in accordance with the Helsinki Declaration and Good Clinical Practice.

### 2.2 Information collection

A questionnaire was completed by all participants, and uniformly trained investigators collected additional demographic information through face-to-face interviews. The collected demographic information included name, sex, date of birth, and educational level. The participants were categorized into four age groups: 60–64, 65–69, 70–74, and ≥75 years. The participants were also classified into three groups according to their total formal education: illiterate (no formal education), primary school (1–6 years), middle school (7–9 years), and high school (>9 years). These education categories were additionally used in sensitivity analyses. Participant personal history included information regarding the presence of hypertension, diabetes mellitus, and lifestyle factors (including smoking and alcohol status). Information was obtained from self-reports and/or existing medical records. Smoking status was categorized as never smoker (never smoked or < 100 cigarettes lifetime), former smoker (previously smoked but not currently smoking), and current smoker (currently smoking within the previous 30 days of survey). Alcohol status was categorized as never drinker (never drank or not drinking at all), former drinker (previously drank but not currently drinking), and current drinker (currently drinking >500 g of alcohol per week for ≥1 year at the time of survey).

### 2.3 Physical examination and biochemical tests

Each participant underwent a physical examination that included measurements of height, weight, blood pressure, and various other physical characteristics. BMI was calculated as weight (kg) divided by height (in meters squared). According to the Chinese BMI classification, BMI was divided into four layers, with underweight defined as < 18.5 kg/m^2^, normal weight as 18.5– < 24 kg/m^2^, overweight as 24– < 28 kg/m^2^, and obesity as ≥28 kg/m^2^ (22). Blood pressure was measured after 5 min of rest in a seated position using an automated sphygmomanometer. The average of two blood pressure measurements was used. Participants with a systolic blood pressure (SBP) ≥140 mmHg, a diastolic blood pressure (DBP) ≥90 mmHg, self-reported history of hypertension or the use of any antihypertensive medication were characterized as having hypertension (23).

Blood samples were collected from all participants after an overnight fast of at least 10 h. We measured levels of FBG, total cholesterol (TC), triglycerides (TG).

### 2.4 Impaired fasting glucose and diabetes

Impaired fasting glucose (IFG) was defined as a FBG of 6.1–6.9 mmol/L. Diabetes was defined as a self-reported diagnosis previously determined by a health care professional, FBG level ≥126 mg/dl, 2-h plasma glucose level ≥200 mg/dl (after an oral dose of 75 g of glucose), or a glycated hemoglobin (HbA1c) level ≥6.5% (24).

### 2.5 Cognitive function screening

Cognitive function was measured by a psychologist using MMSE. It is a widely available cognitive tool used to measure cognitive decline, assessing the following areas: orientation, memory, attention and numeracy, recall, and language skills on a scale of 0–30, with lower scores indicating poorer cognitive function (25). Attending-level psychiatrists collected information on the current state and history of disease, conducted physical examinations, and determined a diagnosis using the Structured Clinical Interview for Diagnostic and Statistical Manual-Fourth edition. Cognitive impairment was defined as an MMSE score of < 18 in the illiterate group, < 21 in the primary school group, and < 25 in the junior school and above groups (26).

Prior to the formal survey, the evaluators underwent a two-day standardized training program: ① Learning the standardized MMSE items and scoring manual. ② Viewing consistency demonstration videos. ③ Conducting a pre-test with 20 non-study participants, with the qualification criterion being an intra-group correlation coefficient (ICC) of 0.90. During the survey, 5% of samples were randomly selected quarterly for dual-evaluator assessment. The study results demonstrated an intra-evaluator ICC of 0.91 (95% confidence interval: 0.87–0.94) and a Kappa value of 0.88, indicating good scoring consistency.

### 2.6 Statistical analysis

This study was a cross-sectional investigation, with cognitive impairment (MMSE cutoff) as the primary outcome measure. Based on previous reports from rural Tianjin, the prevalence of cognitive impairment in elderly individuals with normal blood pressure was approximately 35%. With an allowable error of 5% and a two-tailed alpha level of 0.05, the minimum sample size was calculated using the single-proportion formula *n* = *Z*^2^ × *P* (1–*P*)/ Δ^2^, yielding a minimum sample size of 350 cases. After adjusting for a 10% non-response rate, the required sample size was 389 cases. A total of 386 cases were actually enrolled, which largely met the predefined precision requirements.

All continuous variables, including age; BMI; education; blood pressure; and levels of TC, TG and FBG, are presented as means (standard deviations), and between-group differences were compared using Student's *t*-test or the Mann–Whitney *U*-test. All categorical variables, including sex, age group, education level, smoking status, and alcohol status, are presented as numbers (percentages), and the between-group differences were analyzed using the Chi-squared test. In multivariate modeling, variable selection integrates statistical evidence, clinical relevance, and prior evidence: factors with P-values < 0.5 from univariate analyses, as well as previously established factors related to cognitive function, are incorporated into multivariate analysis. Multivariate logistic regression analysis was used to assess factors associated with cognitive decline. Because dichotomizing MMSE may reduce statistical power, we present logistic models for cognitive impairment and linear models for continuous MMSE as complementary analyses Given the highly skewed distribution of education in this cohort, we did not include *years of education* as a continuous covariate in the primary multivariable models. However, we conducted sensitivity analyses by including education categories to assess the robustness of the findings. Results are presented as adjusted odds ratios (ORs) or β of 95% ci. *A P* < value of 0.05 was statistically significant. Analysis was performed using SPSS version 27.0.

## 3 Results

### 3.1 Participant demographics

This study included 191 male and 195 female normotensive participants. The average age of the participants was 65.88 years. In this study, 23.6% of the participants were illiterate, 9.4% were diabetes, 6.5% of the participants were underweight, 30.8% were overweight, 8.5% were obese and 35.2% had cognitive impairment (Table 1).

**Table 1:** Baseline characteristics of the study population.

**Characteristic**	**Men**	**Women**	**Total**
Case, *n* (%)	191 (49.5)	195 (50.5)	386 (100.0)
Age, years	66.54 (5.74)	65.23 (5.11)	65.88 (5.46)
**Age groups**, ***n*** **(%)**			
60–64 years	90 (47.1)	107 (54.9)	197 (51.0)
65–69 years	54 (28.3)	57 (29.2)	111 (28.8)
70–74 years	25 (13.1)	16 (8.2)	41 (10.6)
≥75 years	22 (11.5)	15 (7.7)	37 (9.6)
Education level, years	5.54 (2.98)	2.96 (3.17)	4.24 (3.33)
**Education groups**, ***n*** **(%)**			
0 year	15 (7.9)	76 (39.0)	91 (23.6)
1–6 years	121 (63.4)	92 (47.2)	213 (55.2)
7–9 years	42 (22.0)	21 (10.8)	63 (16.3)
≥10 years	13 (6.8)	6 (3.1)	19 (4.9)
**Smoking status**, ***n*** **(%)**			
Never smoking	43(22.5)	179(91.8)	222(57.5)
Former smoking	39(20.4)	1(0.5)	40(10.4)
Current smoking	109(57.1)	15(7.7)	124(32.1)
**Alcohol status**, ***n*** **(%)**			
Never drinking	74 (38.7)	185 (94.9)	259 (67.1)
Former drinking	25 (13.1)	2 (1.0)	27 (7.0)
Current drinking	92 (48.2)	8 (4.1)	100 (25.9)
BMI, kg/m^2^	22.97 (3.08)	23.80 (3.35)	23.39 (3.24)
**BMI group**, ***n*** **(%)**			
Underweight	16 (8.4)	9 (4.6)	25 (6.5)
Normal	110 (57.6)	99 (50.8)	209 (54.1)
Overweight	53 (27.7)	66 (33.8)	119 (30.8)
Obesity	12 (6.3)	21 (10.8)	33 (8.5)
**Diabetes groups**, ***n*** (%)^*^			
Normal	157 (82.6)	150 (77.7)	307 (80.2)
Impaired fasting glucose	19 (10.0)	21 (10.9)	40 (10.4)
Diabetes	14 (7.4)	22 (11.4)	36 (9.4)
SBP, mmHg	125.76 (9.75)	126.32 (9.71)	126.04 (9.72)
DBP, mmHg	76.57 (6.83)	74.87 (7.13)	75.71 (7.02)
FBG, mmol/L^*^	5.36 (1.17)	5.58 (1.42)	5.47 (1.31)
TC, mmol/L^*^	4.44 (1.47)	4.81 (1.36)	4.62 (1.42)
TG, mmol/L^*^	1.41 (1.15)	1.54 (0.90)	1.48 (1.03)
MMSE	23.12 (4.71)	20.42 (4.97)	21.75 (5.02)
**Cognitive impairment**, ***n*** **(%)**			
No	137 (71.7)	113 (57.9)	250 (64.8)
Yes	54 (28.3)	82 (42.1)	136 (35.2)

Continuous variables were expressed as mean (standard deviation). BMI body mass index, SBP systolic blood pressure, DBP diastolic blood pressure, FBG fasting blood glucose, TG triglycerides, TC total cholesterol.

^*^represents the deletion value, among which 3 cases were missing FBG, TC and TG.

### 3.2 Factors associated with the cognitive impairment and MMSE scores in the univariate analysis

Univariate analysis showed that there was a significant association between sex, age, smoking status and cognitive impairment (*P* < 0.05) (Table 2). Age, gender, smoking status, alcohol status, and BMI level were correlated with MMSE score (all *P* < 0.05). Specifically, women, older adults over 75 and low BMI were associated with lower MMSE scores (all *P* < 0.05) (Table 3).

**Table 2:** Risk factors associated with cognitive impairment in univariate analysis.

**Characteristic**	**Normal cognition**	**Cognitive impairment**	***T*/*X*^2^**	***P***
Case, *n* (%)	250 (64.8)	136 (35.2)	–	–
Age, years	65.33 (4.95)	66.88 (6.19)	−2.522	0.012
**Gender**, ***n*** **(%)**				
Men	137 (54.8)	54 (39.7)	8.028	0.005
Women	113 (45.2)	82 (60.3)		
**Age groups**, ***n*** **(%)**				
60–64 years	140 (56.0)	57 (41.9)	8.292	0.040
65–69 years	68 (27.2)	43 (31.6)		
70–74 years	23 (9.2)	18 (13.2)		
≥75 years	19 (7.6)	18 (13.2)		
**Smoking status**, ***n*** **(%)**				
Never smoking	133 (53.2)	89 (65.4)	6.334	0.042
Former smoking	91 (36.4)	33 (24.3)		
Current smoking	26 (10.4)	14 (10.3)		
**Alcohol status**, ***n*** **(%)**				
Never drinking	158 (63.2)	101 (74.3)	4.949	0.084
Former drinking	19 (7.6)	8 (5.9)		
Current drinking	73 (29.2)	27 (19.9)		
**Diabetes groups**, ***n*** (%)^*^				
Normal	201 (80.7)	106 (79.1)	0.160	0.923
Impaired fasting glucose	25 (10.0)	15 (11.2)		
Diabetes	23 (9.2)	13 (9.7)		
BMI, kg/m^2^	23.57 (3.25)	23.06 (3.21)	1.481	0.140
**BMI group**, ***n*** **(%)**				
Underweight	16 (6.4)	9 (6.6)	1.192	0.755
Normal	132 (52.8)	77 (56.6)		
Overweight	78 (31.2)	41 (30.1)		
Obesity	24 (9.6)	9 (6.6)		
SBP, mmHg	125.53 (10.01)	126.99 (9.14)	−1.412	0.159
DBP, mmHg	76.15 (7.12)	74.90 (6.78)	1.684	0.093
FBG, mmol/L^*^	5.44 (1.10)	5.53 (1.62)	−0.653	0.514
TC, mmol/L^*^	4.60 (1.41)	4.66 (1.44)	−0.343	0.732
TG, mmol/L^*^	1.51 (1.09)	1.41 (0.91)	0.864	0.388

Continuous variables were expressed as mean (standard deviation). BMI, body mass index; SBP, systolic blood pressure; DBP, diastolic blood pressure; FBG, fasting blood glucose; TG, triglycerides; TC, total cholesterol.

^*^represents the deletion value, among which 3 cases were missing FBG, TC and TG.

**Table 3:** Risk factors associated with MMSE scores in univariate analysis.

**Characteristic**	**Reference**	**B(95%CI)**	***P***
**Gender**	Men	–	–
	Women	−2.70 (−3.66 –−1.73)	< 0.001
Age – years	–	−0.17 (−0.26 –−0.08)	< 0.001
**Age groups**	60–64 years	–	–
	65–69 years	−0.34 (−1.50 – 0.82)	0.567
	70–74 years	−0.92 (−2.60 – 0.75)	0.280
	≥75 years	−3.02 (−4.77 –−1.28)	< 0.001
**Smoking status**	Never smoking	–	–
	Current smoking	2.13 (1.05 – 3.22)	< 0.001
	Former smoking	2.27 (0.61 – 3.93)	0.008
**Alcohol status**	Never drinking	–	–
	Current drinking	2.08 (0.94 – 3.22)	< 0.001
	Former drinking	2.80 (0.84 – 4.75)	0.005
**Diabetes groups** ^*^	Normal	–	–
	Impaired fasting glucose	−1.18 (−2.84 – 0.48)	0.163
	Diabetes	−0.93 (−2.67 – 0.82)	0.297
BMI, kg/m^2^	–	0.16 (0.01 – 0.32)	0.042
**BMI group**	Normal	–	–
	Underweight	0.26 (0.81 –−2.35)	0.805
	Overweight	0.48 (−0.65 – 1.61)	0.405
	Obesity	1.41 (−0.44 – 3.26)	0.135
SBP, mmHg	–	−0.04 (−0.09 −0.01)	0.129
DBP, mmHg	–	0.04 (−0.03 – 0.11)	0.250
FBG, mmol/L^*^	–	−0.22 (−0.60 – 0.17)	0.273
TC, mmol/L^*^	–	−0.22 (−0.58 – 0.14)	0.225
TG, mmol/L^*^	–	0.00 (−0.49 – 0.49)	0.999

MMSE, mini-mental state examination; BMI, body mass index; SBP, systolic blood pressure; DBP, diastolic blood pressure; FBG, fasting blood glucose; TG, triglycerides; TC, total cholesterol; β, regression coefficient; 95% CI confidence interval.

^*^represents the deletion value, among which 3 cases were missing FBG, TC and TG.

### 3.3 Factors associated with cognitive impairment and MMSE scores in the multivariate analysis

The factors of *P* < 0.5 in univariable analyses and clinically relevant covariates were entered into the multivariate logistic analysis, multivariate analysis showed that there was no statistically significant association between these factors and cognitive impairment (*P* all >0.05) (Table 4). Table 5 shows the results of multivariate analysis related to MMSE scores. After adjusting for sex, age groups, smoking status, alcohol status, BMI levels, the results showed that sex, age group, BMI levels were independent factors influencing the MMSE score (*P* all < 0.05). Compared with men, the MMSE score of women decreased by 2.40 points (β = −2.40, 95%CI: −3.78 – −1.02, *P* < 0.001), and compared with 60–64 years old, the MMSE scores of people over 75 years old decreased by an average of 3.18 points (95%CI: −4.88 – −1.49, *P* < 0.001), and the MMSE score of people 70–74 years old increased by 0.12 points (95%CI: −2.66 – 0.62, *P* = 0.221).

**Table 4:** Risk factors associated with cognitive impairment in multivariate analysis.

**Risk factors**	**Reference**	**OR (95%CI)**	***P***
**Age groups**	60–64 years	–	–
	65–69 years	1.48 (0.88 – 2.47)	0.138
	70–74 years	1.81 (0.85 – 3.84)	0.123
	≥75 years	2.06 (0.96 – 4.45)	0.065
**Gender**	Men	–	–
	Women	1.70 (0.91 – 3.19)	0.098
**Alcohol status**	Never drinking	–	–
	Current drinking	0.85 (0.42 – 1.72)	0.650
	Former drinking	1.25 (0.49 – 3.23)	0.641
**Smoking status**	Never smoking	–	–
	Current smoking	0.75 (0.37 – 1.52)	0.422
	Former smoking	1.25 (0.49 – 3.23)	0.641
BMI, kg/m^2^	–	0.94 (0.87 – 1.01)	0.105
SBP, mmHg	–	1.03 (1.00 – 1.06)	0.051
DBP, mmHg	–	0.97 (0.94 – 1.01)	0.172
TG, mmol/L	–	0.97 (0.77 – 1.24)	0.826

OR odds ratio, 95% CI confidence interval.

Three cases with TG deletion were excluded, and a total of 383 people were included in the multivariate analysis.

**Table 5:** Risk factors associated with MMSE scores in multivariate analysis.

**Characteristic**	**Reference**	**β (95% CI)**	***P***
**Gender**			
Men	–	–	–
Women	–	−2.40 (−3.78 -−1.02)	< 0.001
**Age groups**			
60–64 years	–	–	–
65–69 years	–	−0.39 (−1.50 – 0.72)	0.489
70–74 years	–	−1.02 (−2.66 – 0.62)	0.221
≥75 years	–	−3.18 (−4.88 –−1.49)	< 0.001
**Smoking status**			
Never drinking	–	–	–
Current smoking	–	0.45 (−1.08 – 1.98)	0.565
Former smoking	–	0.23 (−1.84 – 2.30)	0.828
**Alcohol consumption**			
Never smoking	–	–	–
Current drinking	–	0.49 (−0.98 – 1.95)	0.487
Former drinking	–	1.10 (−1.08 – 3.28)	0.322
BMI, kg/m^2^	–	0.18 (0.03 – 0.33)	0.019

MMSE, mini-mental state examination; FBG, fasting blood glucose; β, regression coefficient; 95% CI confidence interval.

A total of 386 people were included in the multivariate analysis.

### 3.4 Subgroup analysis

We further explored the influencing factors of cognitive impairment and MMSE score in gender and age subgroups (Tables 6, 7), and the results showed that the risk of cognitive impairment in women aged 65–69 years was 2.09 times higher than that in people aged 60–64 years (OR: 2.09, 95%CI: 1.07 – 4.09, *P* = 0.031). Among 65–69 year olds, the risk of cognitive impairment increased by 5% for each additional unit of SBP (OR: 1.05, 95%CI: 1.00 – 1.10, *P* = 0.041). Among people aged 70–74 years, the risk of cognitive impairment decreased by 82% for every 1 unit increase in TG (OR: 0.18, 95%CI: 0.04 – 0.94, *P* = 0.042). Among people over 75 years of age, the risk of cognitive impairment increased by 991% for each unit increase in TG (OR: 10.91, 95%CI: 1.20 – 99.44, *P* = 0.034), and alcohol status may be a protective factor for cognition (*P* < 0.05). However, the subgroup analysis results are exploratory in nature, with some factors exhibiting opposite effects across groups and wide confidence intervals, warranting cautious interpretation of the findings.

**Table 6:** Multivariate analysis of the number of cognitive impairment in subgroups by gender and age.

**Risk factors**	**Reference**	**OR (95%CI)**	***P***
**Men**			
Age groups	60–64 years	–	–
	65–69 years	0.95 (0.42 – 2.19)	0.912
	70–74 years	1.24 (0.44 – 3.49)	0.680
	≥75 years	1.57 (0.54 – 4.57)	0.406
**BMI group**			
Underweight	–	–	–
Normal	–	1.47 (0.45 – 4.80)	0.524
Overweight	–	0.49 (0.13 – 1.90)	0.303
Obesity	–	0.25 (0.02 – 1.01)	0.266
**Smoking status**			
Never drinking	–	–	–
Current smoking	–	0.53 (0.23 – 1.20)	0.128
Former smoking	–	1.36 (0.51 – 3.65)	0.543
TG, mmHg	–	0.98 (0.68 – 1.41)	0.902
DBP, mmHg	–	0.96 (0.91 – 1.01)	0.122
**Women**			
Age groups	60–64 years	–	–
	65–69 years	2.09 (1.07 – 4.09)	0.031
	70–74 years	2.19 (0.71 – 6.76)	0.173
	≥75 years	2.32 (0.71 – 7.59)	0.163
**Smoking status**			
Never smoking	–	–	–
Current smoking	–	1.80 (0.46 – 7.00)	0.395
Former smoking	–	–	–
**Alcohol status**			
Never drinking	–	–	–
Current drinking	–	0.58 (0.10 – 3.42)	0.549
Former drinking	–	–	–
SBP, mmHg	–	1.03 (0.99 – 1.06)	0.134
**60–64 years**			
Smoking status	Never smoking	–	–
	Current smoking	0.62 (0.29 – 1.31)	0.618
	Former smoking	1.71 (0.58 – 5.10)	0.334
Diabetes groups	Normal	–	–
	Impaired fasting glucose	0.30 (0.06 – 1.45)	0.134
	Diabetes	1.08 (0.38 – 3.08)	0.881
Age, years	–	0.82 (0.65 – 1.03)	0.089
BMI, kg/m^2^	–	0.93 (0.83 – 1.04)	0.202
SBP, mmHg	–	1.00 (0.96 – 1.04)	0.853
DBP, mmHg	–	0.98 (0.93 – 1.04)	0.545
Smoking status	Never smoking	–	–
	Current smoking	0.35 (0.10 – 1.30)	0.353
	Former smoking	0.21 (0.03 – 1.68)	0.214
Alcohol status	Never drinking	–	–
	Current drinking	0.85 (0.23 – 3.21)	0.849
	Former drinking	2.72 (0.37 – 19.84)	0.323
Diabetes groups	Normal		
	Impaired fasting glucose	2.11 (0.53 – 8.45)	0.293
	Diabetes	0.99 (0.14 – 7.08)	0.995
TC, mmol/L	–	1.13 (0.73 – 1.76)	0.589
SBP, mmHg	–	1.05 (1.00 – 1.10)	0.041
**70–74 years**			
Age, years	–	1.24 (0.76 – 2.03)	0.394
BMI, kg/m^2^	–	1.13 (0.83 – 1.54)	0.444
DBP, mmHg	–	0.96 (0.56 – 1.08)	0.511
TG, mmol/L	–	0.18 (0.04 – 0.94)	0.042
Alcohol status	Never drinking	–	–
	Current drinking	0.42 (0.05 – 3.17)	0.397
	Former drinking	–	–
≥**75 years**			
Alcohol status	Never drinking	–	–
	Current drinking	0.08 (0.01 – 0.84)	0.036
	Former drinking	0.97 (0.04 – 24.49)	0.985
Diabetes groups	Normal	–	–
	Impaired fasting glucose	1.20 (0.14 – 10.09)	0.865
	Diabetes	0.55 (0.02 – 13.26)	0.710
Age, years	–	1.14 (0.86 – 1.51)	0.374
TC, mmol/L	–	0.56 (0.20 – 1.59)	0.276
TG, mmol/L	–	10.91 (1.20 – 99.44)	0.034
SBP, mmHg	–	1.10 (0.97 – 1.25)	0.145

The factors of P < 0.5 in the univariate analysis results related to cognitive impairment were included in the multivariate logistic analysis.

One case with TG deletion was excluded from the male subgroup, leaving a total of 190 individuals. The female subgroup comprised 195 individuals. For age stratification, the 60–64 years subgroup included 197 individuals; the 65–69 years subgroup included 110 individuals (with 1 case of TC deletion); the 70–74 years subgroup consisted of 39 individuals (with 2 cases of TG deletion); and the ≥75 years subgroup comprised 37 individuals.

**Table 7:** Multivariate analysis of the number of MMSE in subgroups by gender and age.

**Risk factors**	**Reference**	**β (95%CI)**	***P***
**Man:**			
Age, years	–	−0.10 (−0.21 – 0.02)	0.106
BMI, kg/m^2^	–	0.37 (0.16 – 0.59)	< 0.001
DBP, mmHg	–	0.06 (−0.04 – 0.16)	0.224
Smoking status	Never smoking	–	–
	Current smoking	1.31 (−0.32 – 2.94)	0.116
	Former smoking	0.70 (−1.27 – 2.67)	0.485
**Woman:**			
Age, years	–	−0.25 (−0.38 –−0.11)	< 0.001
SBP, mmHg	–	−0.07 (−0.14 – 0.00)	0.054
**60–64 years:**			
Smoking status	Never smoking	–	–
	Current smoking	2.41 (1.00 – 3.81)	< 0.001
	Former smoking	1.22 (-1.03 – 3.46)	0.287
**65–69 years:**			
Smoking status	Never smoking	–	–
	Current smoking	3.11 (1.04 – 5.17)	0.004
	Former smoking	2.58 (−0.74 – 5.91)	0.126
**70–74 years:**			
Alcohol status	Never drinking	–	–
	Current drinking	2.29 (−1.10 – 5.68)	0.180
	Former drinking	5.95 (0.57 – 11.34)	0.031
≥**75 years:**			
Diabetes groups	Normal	–	–
	Impaired fasting glucose	−1.94 (−6.59 – 2.72)	0.402
	Diabetes	−2.63 (−9.66 – 4.40)	0.451
Alcohol status	Never drinking	–	–
	Current drinking	5.01 (0.88 – 9.14)	0.019
	Former drinking	1.60 (−7.18 – 10.39)	0.712
TG, mmol/L	–	−3.39 (−7.32 – 0.54)	0.088

The factors of P < 0.05 in the univariate analysis results related to MMSE were included in the multivariate logistic analysis.

The male subgroup comprised 191 individuals, and the female subgroup comprised 195 individuals. For age stratification, the 60–64 years, 65–69 years, 70–74 years, and ≥75 years subgroups included 197, 111, 41, and 37 individuals, respectively.

### 3.5 Sensitivity analysis

In sensitivity analyses, education was additionally included as categorical groups in the multivariable regression models. Education showed an independent association with cognitive impairment. Compared with illiteracy, the odds of cognitive impairment were lower among participants with primary school education (*OR* = 0.38, 95% CI: 0.22 – 0.66; *P* < 0.001) and among those with junior high school education or above (*OR* = 0.29, 95% CI: 0.14 – 0.61; *P* = 0.001). Importantly, after additionally adjusting for education categories, the main associations observed in the primary analyses (e.g., age group, sex, and BMI) remained directionally consistent and were not materially changed, supporting the robustness of our findings (Tables 8, 9).

**Table 8:** Sensitivity analysis of risk factors associated with cognitive impairment in multivariate analysis. (include in education grouping).

**Risk factors**	**Reference**	**OR (95%CI)**	***P***
**Education groups**	0 year	–	–
	1–6 years	0.38 (0.22 – 0.66)	< 0.001
	>6 years	0.29 (0.14 – 0.61)	0.001
**Age groups**	60–64 years	–	–
	65–69 years	1.56 (0.92 – 2.63)	0.100
	70–74 years	1.63 (0.75 – 0.53)	0.215
	≥75 years	–	–
**Gender**	Men	1.22 (0.63 – 2.39)	0.556
	Women	–	–
**Alcohol status**	Never drinking	–	–
	Current drinking	0.90 (0.44 – 1.83)	0.763
	Former drinking	1.04 (0.36 – 3.06)	0.939
**Smoking status**	Never smoking	–	–
	Current smoking	0.76 (0.37 – 1.56)	0.448
	Former smoking	1.26 (0.48 – 3.31)	0.640
BMI, kg/m^2^	–	0.94 (0.88 – 1.02)	0.139
SBP, mmHg	–	0.96 (0.75 – 1.22)	0.723
DBP, mmHg	–	0.97 (0.93 – 1.01)	0.092
TG, mmol/L	–	0.96 (0.75 – 1.22)	0.723

OR odds ratio, 95% CI confidence interval.

Three cases with TG deletion were excluded, and a total of 383 people were included in the multivariate analysis.

**Table 9:** Sensitivity analysis of risk factors associated with MMSE scores in multivariate analysis. (include in education grouping).

**Characteristic**	**Reference**	**β (95% CI)**	***P***
Education groups	1–6 years	3.36 (2.32 – 4.41)	< 0.001
	>6 years	6.35 (4.92 – 7.77)	< 0.001
Gender	Men	–	–
	Women	−1.42 (−2.70 –−0.13)	0.031
Age groups	60–64 years	–	–
	65–69 years	−0.62 (−1.64 – 0.39)	0.227
	70–74 years	−0.76 (-2.24 – 0.73)	0.319
	≥75 years	−2.07 (−3.63 –−0.51)	0.009
Smoking status	Never smoking	–	–
	Current smoking	0.38 (−1.01 – 1.77)	0.594
	Former smoking	0.12 (−1.77 – 2.01)	0.898
Alcohol status	Never drinking	–	–
	Current drinking	0.07 (−1.27 – 1.40)	0.920
	Former drinking	0.38 (−1.62 – 2.38)	0.708
BMI, kg/m^2^	–	0.19 (0.05 – 0.33)	0.007

MMSE Mini-Mental State Examination, β regression coefficient, 95% CI confidence interval.

A total of 386 people were included in the multivariate analysis.

## 4 Discussion

Our study aimed to investigate the cognitive function status and related risk factors for normotensive elderly individuals. The study found that approximately 35.2% of the elderly participants experienced cognitive impairment despite having normal blood pressure. Significant risk factors associated with lower MMSE scores included older age, female gender, and low BMI levels. Multivariate analysis showed that age, gender, and low BMI levels remained independent predictors of cognitive function. This study highlighted that even in normotensive individuals, other factors such as low BMI levels and gender play significant roles in cognitive function. Additionally, the finding that FBG levels, alcohol status and smoking status were less strongly associated with cognitive impairment in this population contrasts with results from studies on hypertensive or general elderly populations. Notably, multivariate logistic regression failed to identify significant independent predictors for classified cognitive impairment, whereas linear regression demonstrated significant associations between age, gender, BMI, and MMSE. This discrepancy primarily stems from the dichotomization method's reduced test power due to cut-off point selection and information loss, whereas treating MMSE as a continuous variable may better capture subtle cognitive differences. Given the well-known influence of education on cognitive test performance, we performed sensitivity analyses additionally adjusting for education categories. The main associations (age, sex, and BMI) were largely unchanged, supporting the robustness of our primary findings.

The prevalence of dementia and cognitive impairment is not uniform in different countries and regions. In the United States, the estimated prevalence of dementia among people older than 65 years was 10%, and the prevalence of mild cognitive impairment was 22% in 2016 (2). The prevalence of dementia in India was estimated to be 7.4% among adults aged over 60 years (27). In rural areas of northern China, the prevalence of dementia, Alzheimer's Disease and vascular dementia among adults aged over 60 years was 7.7%, 5.4% and 1.7%, respectively (28). In our previous study, the prevalence of cognitive impairment in the elderly without cardiovascular and cerebrovascular diseases was 32.4% (29). However, few studies have reported the prevalence of cognitive impairment in normotensive, low-income older adults. In this study, there was 35.2% of the normotensive elderly participants experienced cognitive impairment. On the one hand, the high prevalence in this population may be explained by the low level of education. On the other hand, the diagnostic criteria for cognitive impairment in this study were relatively loose, and a large proportion of subjects diagnosed with cognitive impairment did not meet the criteria for dementia according to the Diagnostic and Statistical Manual of Mental Disorders IV (DSM-IV).

Age as an independent risk factor for cognitive impairment is well established. A US cross-sectional study showed that lower MoCA scores are associated with older age (30). In addition, other previously published findings on cognitive decline show similar results (28, 29, 31–33). And a study that tracked changes in cognitive health between the ages of 55 and 95 found that many of the cognitive declines didn't show up until after 75 years old (33). Similarly, our study found that age remained significant predictors of cognitive function in normotensive older adults. The possible mechanism is that cortical inputs to the hippocampal system are impaired with age, resulting in cognitive decline, particularly in episodic memory (34). In addition, age-related changes in brain structure and cognition may be associated with leukocyte telomere length changes (35).

Gender differences in cognitive impairment are still controversial. Some studies suggest that male gender is a risk factor for cognitive impairment (30, 36). A study of older US adults living at home showed that women had higher MoCA scores than men at age 62, but the two were nearly equal by age 90 (30). Other studies have found a significantly higher prevalence of cognitive impairment in women than in men (28, 31, 37). Yong Ji et al. found that female gender was a risk factor for Alzheimer's disease, but not for vascular dementia (28). In our study, female gender was associated with lower MMSE scores. This difference may be attributed to the different populations and regions studied, and future multicenter studies are needed to confirm it.

BMI level is also a significant correlation factor for cognitive impairment. A cross-sectional study in China suggests that being overweight is associated with a reduced risk of cognitive impairment in older Chinese adults. (38) Underweight has been identified in prospective studies as a risk factor for cognitive impairment, especially in older women (17). In addition, greater BMI variability, greater weight gain, or substantial weight loss were associated with increased risk (39, 40). A 10-year prospective study showed differences in the impact of BMI on cognition among older adults of different ages, with higher BMI before age 65 being associated with lower cognitive ability, a faster rate of cognitive decline, and a higher chance of cognitive impairment later in life; After age 65, the association flipped, with higher BMI associated with better late-life cognitive outcomes. And the correlation was stronger for women at both ages (21). Similarly, we also found that low BMI levels were associated with a higher risk of cognitive impairment, but in our study this association was particularly strong in men and may be related to selected population characteristics that warrant further study. Furthermore, this study is a cross-sectional study and cannot establish causal relationships. This association may be due to reverse causality, such as cognitive decline potentially promoting weight loss. Future validation of these conclusions requires more large-sample prospective studies.

Cognitive impairment is a recognized complication associated with diabetes, but there are differences in research results regarding whether blood glucose levels are related to cognitive dysfunction. Some studies support a positive correlation between blood glucose levels and cognitive impairment (41–43). A prospective study in China found that participants with type 2 diabetes mellitus (T2DM) with high FBG levels had the highest cumulative incidence of dementia, compared with non-diabetic participants and participants with low FBG levels, and that baseline FBG levels were positively associated with the annual rate of MMSE decline participants with T2DM (41). In addition, a prospective study in US confirmed that higher average blood glucose levels were associated with an increased risk of dementia, both in diabetic and non-diabetic participants (42). Elevated blood glucose levels may contribute to an increased risk of dementia and cognitive decline through several potential mechanisms. On the one hand, cognitive decline mediated by high blood glucose levels may be related to neuroinflammation of brain tissue (43). Studies have shown that high blood glucose levels can damage the blood-brain barrier and cause neuroinflammation in the brain tissue, possibly leading to cognitive impairment (44, 45). Studies on neuroinflammatory and molecular indicators of early cognitive impairment in T2DM mouse models have shown that cognitive impairment is not only related to increased cytokine levels but also to decreases in *Arc* and *Egr1* mRNA expression levels in the brain regions related to learning processes and memory formation (46). On the other hand, high blood sugar may mediate cognitive decline by altering synaptic function. In mouse models, hyperglycemia exposure may lead to abnormal synaptic plasticity (47). And synaptic loss is known to be a major factor associated with cognitive decline in humans (48). However, some other studies have come to different conclusions about blood glucose levels and cognitive function. An analysis of data from two independent prospective studies concluded that elevated FBG levels and insulin resistance were not associated with worsening cognitive function in older adults without diabetes (15). This suggests that there may be a threshold for the effect of abnormal blood glucose on cognitive function. Liu et al. found that increased FBG levels in diabetic individuals were associated with decreased cognitive function, but for non-diabetic individuals, there was an inverse U-shaped relationship between FBG levels and cognitive function, and the cognitive function score was highest when fasting glucose was 3.97–6.20 mmol/L (49). And another possibility is that factors other than abnormal blood glucose may cause cognitive impairment in diabetic patients. In addition, Yuan, Z. et al study did not find an association between FBG or diabetes and cognition in middle-aged and older adults (50). Our study also did not find a direct association between FBG or diabetes and cognitive impairment. In elderly people with normal blood pressure, whether high FBG level is still an influential factor for cognitive impairment deserves further verification.

Previous studies have shown that smoking and heavy alcohol status may impair cognitive function. Smoking increases the risk of dementia and cognitive decline in older adults (51, 52). Recent studies have shown that environmental tobacco smoke (ETS), also known as secondhand or passive smoking, is also considered a risk factor for dementia syndrome, which is related to the level and duration of exposure (53). Exposure to cigarette smoke may exacerbate cognitive impairment through neuroinflammation (54). The association between alcohol status and dementia was heterogeneous. Long-term heavy alcohol status may have a negative impact on cognitive function, while light to moderate consumption does not (55). In addition, Manja Koch et al. found that daily light alcohol status was associated with a lower risk of dementia compared to infrequent heavy alcohol status (56). However, in our study among older normotensive population, alcohol status and smoking status were weakly associated with cognitive impairment. However, subgroup analysis in this study revealed that alcohol status provided cognitive protection in individuals aged ≥75 years, contradicting previous findings. This discrepancy may arise because the potential cognitive benefits of moderate alcohol intake in older adults might be mediated through mechanisms such as improved vascular endothelial function, elevated high-density lipoprotein (HDL) levels, or enhanced cerebral blood flow. Nevertheless, given the cross-sectional design of this study, we cannot rule out health survivor bias or reverse causality (i.e., cognitively intact individuals may be more likely to maintain moderate alcohol status). Therefore, these results should be regarded as hypothesis generation and require validation through prospective cohort studies with larger sample sizes.

### 4.1 Limitations and strengths

This study has some limitations. First, this was a small cross-sectional study, and we could not determine a causal relationship between the identified factors and presence or development of cognitive impairment. The factors identified in this study that are associated with cognitive function may exhibit a reverse causal relationship with cognitive function, suggesting that cognitive impairment could potentially lead to the occurrence of these factors. More large-scale prospective studies are needed to confirm the causal association. One of the important reasons for the small sample size was the high prevalence of hypertension among elderly adults in this area. However, the prevalence of cognitive decline in normotensive elderly individuals is high, accentuating the need to study the risk factors of cognitive function in this population. Second, data on smoking and alcohol status were collected only as categorical information (never, previously, currently), without recording daily cigarette count, pack-years, alcohol type, or ethanol grams. Consequently, dose-response relationships could not be evaluated, nor could residual confounding due to exposure intensity or duration be excluded. Future studies should employ standardized questionnaires (e.g., WHO Alcohol Use Disorder Screening Scale, Fagerstrom Nicotine Dependence Scale) to collect pack-years, standard drinking units, and lifetime exposure trajectories. Additionally, factors such as age at initiation/cessation of smoking/alcohol and duration of abstinence should be considered to accurately characterize the dose-response curve of cognitive impairment using methods like trend testing or restricted cubic splines. Third, the study population consisted of low-income individuals in rural northern China, with generally low educational attainment (95.1% of the population had ≤ 9 years of education). Therefore, we included education level only in sensitivity analyses rather than as a primary factor. However, this may have introduced potential confounding effects. Given these limitations, we plan to conduct future prospective studies with larger sample sizes and broader populations to further elucidate the causal relationship between educational attainment and the incidence of cognitive impairment in this population. Fourth, the sample size for subgroup analysis in this study was small, with confidence intervals for certain associations (e.g., the inverse effect of triglycerides across different age groups) being extremely wide. Moreover, the same factor exhibited opposite effects in different subgroups, indicating limited statistical power of the subgroup analysis in this study. The results are exploratory and should be interpreted with caution, requiring validation through large-sample prospective cohort studies. In addition, the sample of this study was only from rural areas in Tianjin, which has geographical limitations. Compared with other regions, there may be differences in socioeconomic development levels, dietary structure, and climate environment in this area, thereby limiting the generalizability of the study results. Future research should conduct multicenter studies among rural populations in the eastern, central, and western regions of China to verify whether these findings apply to elderly populations with different geographical and cultural backgrounds. Finally, subgroup analyses were exploratory and involved multiple comparisons, therefore, the subgroup findings should be interpreted cautiously and considered hypothesis-generating; and we did not perform formal interaction testing (e.g., TG × age group) nor apply multiple-comparison adjustment, which may increase the risk of chance findings.

This study has several strengths. First, it is based on a large-scale population sample of elderly individuals from rural China, providing a comprehensive understanding of cognitive function in this specific demographic. The findings are particularly valuable for understanding cognitive health in rural, low-income areas. Additionally, by focusing on normotensive elderly adults, the study effectively eliminates the confounding effects of hypertension, enabling a more accurate assessment of cognitive function in individuals without common comorbidities associated with cognitive decline. The multidisciplinary approach, integrating epidemiological, neurological, and sociological methods, further enhances the applicability of the findings and offers a broader perspective on factors influencing cognitive health. Finally, the study provides a thorough analysis of various potential contributing factors, such as socioeconomic status, lifestyle behaviors, and health conditions, offering valuable insights for future interventions aimed at improving cognitive health in elderly populations.

## 5 Conclusion

This study found that old age, female gender, and decreased BMI levels are associated with a decrease in MMSE scores in normotensive elderly individuals. These findings suggest that even older adults with normal blood pressure are at a high risk for cognitive impairment. Older men with low BMI and elderly female may need to pay more attention to cognitive issues. Furthermore, community-based screening and education programs targeting low-weight elderly populations may facilitate early detection of cognitive changes; however, longitudinal study evidence is still required before concluding that such interventions can reduce cognitive impairment or its societal burden.

## Data Availability

The raw data supporting the conclusions of this article will be made available by the authors, without undue reservation.
